# Enhanced interleukin-1β production of PBMCs from patients with gout after stimulation with Toll-like receptor-2 ligands and urate crystals

**DOI:** 10.1186/ar3898

**Published:** 2012-07-04

**Authors:** Eleni E Mylona, Maria Mouktaroudi, Tania O Crisan, Stamatoula Makri, Aikaterini Pistiki, Marianna Georgitsi, Athina Savva, Mihai G Netea, Jos WM van der Meer, Evangelos J Giamarellos-Bourboulis, Leo AB Joosten

**Affiliations:** 1 4th Department of Internal Medicine, Attikon University Hospital, 1 Rimini Str., Athens 12462, Greece; 2 Department of Medicine, Radboud University Nijmegen Medical Centre, Geert Grooteplein zuid 8, 6525 GA Nijmegen, The Netherlands; 3 Nijmegen Institute for Infection, Inflammation and Immunity (N4i), 6525 GA Nijmegen, The Netherlands

## Abstract

**Introduction:**

Monosodium urate monohydrate (MSU) crystals synergize with various toll-like receptor (TLR) ligands to induce cytokine production via activation of the NOD-like receptor (NLR) family, pyrin domain-containing 3 (NLPR3) inflammasome. This has been demonstrated *in vitro *using human cell lines or monocytes of healthy volunteers. In the present study, we have investigated the effect of MSU crystals and of their combination with TLR ligands in peripheral blood mononuclear cells (PBMC) of patients with gout.

**Methods:**

PBMCs from 18 patients with primary gout and 12 healthy donors were exposed to MSU crystals in the presence or absence of saturated fatty acid C18:0 (free fatty acid, TLR2 ligand), palmitoyl-3-cystein (Pam_3_Cys, TLR1/2 ligand) and fibroblast stimulating factor-1 (FSL-1, TLR 2/6 ligand). Production of IL-1β, IL-6, IL-8, IL-17 and tumor necrosis factor alpha (TNFα) was determined by ELISA. mRNA transcripts of IL-1β were measured by real-time PCR.

**Results:**

MSU crystals alone failed to induce IL-1β, IL-6 or TNFα in both patients and control groups, but a stronger synergy between MSU/Pam_3_Cys and MSU/C18:0 for the induction of IL-1β was found in patients with gout compared to healthy controls. IL-6, but not IL-8, followed the kinetics of IL-1β. No production of the neutrophil-recruiting IL-17 was detectable after stimulation of the patients' PBMCs with MSU in both the presence or absence of TLR ligands. No change of gene transcripts of IL-1β after stimulation with MSU and Pam_3_Cys or with MSU and C18:0 was found. A positive correlation was found between synergy in IL-1β production from PBMCs of patients between C18:0 and MSU crystals, as well as the annual number of attacks of acute gouty arthritis (r_s_: +0.649, *P*: 0.022).

**Conclusions:**

The synergy between MSU crystals and TLR-2 ligands is more prominent in patients with gout than in controls. This is likely mediated by the enhanced maturation of pro-IL-1β into IL-1β.

## Introduction

Gout is a crystal-induced inflammatory disease induced by the deposition of crystals of monosodium urate monohydrate (MSU) in the joints and in the synovial membranes [1]. The arthritis is mediated by pro-inflammatory cytokines, produced by activated innate immune cells [2]. Among these cytokines, IL-1β seems to play a pivotal role [3]. This was proven in clinical studies, in which selective blockade of IL-1β effectively suppressed pain and inflammation in patients with gout that was refractory to other treatments [4-6].

Synthesis of bioactive IL-1β is induced in two steps: in the first step, gene expression leads to synthesis of inactive pro-IL-1β; in the second step, pro-IL-1β is cleaved by the protease caspase-1 to yield mature IL-1β [7]. For activation of caspase-1, intracellular molecular platforms called inflammasomes have to be assembled [8]. MSU crystals have been reported to activate the MOD-like receptor (NLR) family, pyrin domain-containing 3 (NLPR3) inflammasome and induce IL-1β production via caspase-1-dependent processing [9,10]. However, purified MSU crystals induce moderate amounts of IL-1β by themselves [11,12]; they require co-stimulation with toll-like receptor (TLR) ligands such as lipopolysaccharide (LPS) [11] or free fatty acids (FFA) [12].

This necessity of co-stimulation is in agreement with the clinical wisdom that, in the context of chronically elevated uric acid concentrations, attacks of gout are preceded by infections or excess intake of alcohol or dietary fat.

The *in vitro *studies performed until now have either studied human monocyte cell lines [9] or primary mononuclear cells of healthy volunteers [9,11,12] but the IL-1β production capacity of primary cells of patients with gout has not been investigated. In this study, we investigated whether primary cells [peripheral blood mononuclear cells (PBMCs)] of patients with gout also need this dual stimulation for cytokine production. The role of IL-1β stimulation in gout was investigated in an effort to decipher if the pathophysiological phenomena of attacks of acute gouty arthritis are related to the excess production of IL-1β or of other cytokines mediating chemotaxis of neutrophils at the inflamed synovium, namely IL-18 and IL-17.

## Materials and methods

### Patients

Eighteen patients with gout (13 men, five women, aged 63.5 ± 13.6 years, Table 1) and 12 healthy volunteers (6 men, 6 women, aged 41.17 ± 14.41 years) were asked to donate blood. The study protocol received approval from the Ethics Committee of ATTIKON University Hospital. Patients were enrolled after written informed consent. Inclusion criteria were: a) diagnosis of primary gout; b) history of at least two acute gouty attacks; and c) blood sampling in interictal periods. Patients taking any anti-inflammatory medication in the previous 15 days were excluded from the study. Primary gout and an acute attack of gout were defined according to the criteria outlined by the American Rheumatism Association [13]. After informed consent, 30 ml of venous blood was collected under sterile conditions.

**Table 1:** Clinical characteristics of 18 patients with gout enrolled in the study.

No	Age (years)	Co-morbidities	Number of affected joints	Number of annual attacks	Therapy of attacks
1	75	DM2, VH, COPD	2	2	Colchicine
2	80	DM2, VH, CHD	4	5	Colchicine
3	62	DM2, VH, obesity	1	1	Colchicine
4	80	DM2, COPD, CHD	1	1	Colchicine
5	45	VH, obesity	1	1	Colchicine
6	70	VH	1	1	Colchicine
7	67	DM2, VH, CHD	7	5	NSAIDs
8	65	VH, obesity	1	1	Colchicine
9	62	VH	1	1	Colchicine
10	55	VH, obesity	2	1	Colchicine
11	80	DM2, VH	2	2	NSAIDs
12	32	Obesity	4	6	NSAIDs
13	70	VH	1	1	Colchicine
14	70	DM2, VH	3	2	Colchicine
15	68	Obesity	1	3	Colchicine
16	52	DM2, CRD	1	10	Colchicine
17	69	CRD	5	4	Colchicine
18	46	CRD	1	5	Colchicine

CHD, coronary heart disease; COPD, chronic obstructive pulmonary disease; CRD, chronic renal disease; DM2, diabetes mellitus type 2; NSAIDs, non-steroidal anti-inflammatory drugs; VH, vascular hypertension.

### Preparation of monosodium urate monohydrate (MSU) crystals and of ultrapure C18:0

**Figure 1:**
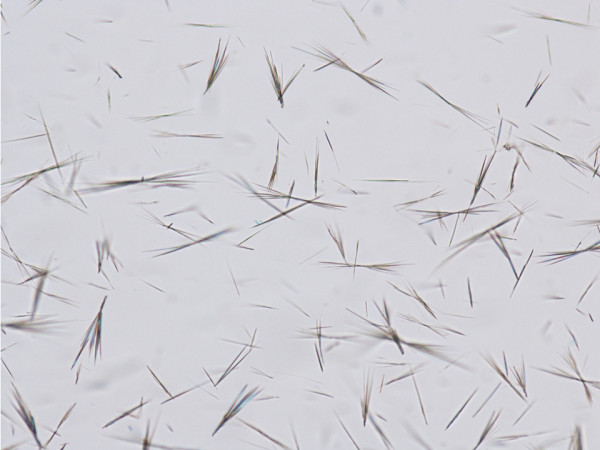
**Polarized microscopy of the prepared crystals of monosodium urate**.

MSU crystals were prepared according to the method described by Seegmiller *et al*. [14]. Briefly, a solution of 0.03 M of MSU at a volume of 200 ml was prepared after diluting 1.0 g of uric acid (Sigma Co, St Louis, MO, USA) in 200 ml of sterile water containing 24 g of NaOH. The pH was adjusted to 7.2 after addition of HCl and the solution became pyrogen-free after incubation for six hours at 120°C. Concentrations of LPS were below 0.01 ng/ml as measured by the kinetic LAL QCL-1000 assay (BioWhittaker-Walkersville, Maryland. USA). The solution was left to cool at room temperature and stored at 4°C. Produced crystals were 5 to 25 μm long (Figure 1). On each day of the experiment, a small amount of crystals was weighted under sterile conditions for application.

A 200 mM stock solution of ultrapure C18:0 fatty acids (Sigma Co) at a volume of 5 ml ethanol 100% was prepared. The stock was warmed at 37°C in a water bath and pre-diluted to 20 mM with ethanol 100%, before use. A concentration of 200 μM C18:0 was used to stimulate PBMCs and the final concentration of ethanol in the wells of a 96-well plate was 0.55 %. Although this concentration appears high, it is consistent with the concentrations that exist in the joint as reflected indirectly by the circulating concentrations of free fatty acids of patients before and after total hip arthroplasty [15].

### Stimulation of cytokine production

PBMCs were isolated after gradient centrifugation of heparinized whole blood over FicolHypaque (Biochrom, Berlin, Germany). After three consecutive washings in ice-cold PBS pH 7.2 (Biochrom), PBMCs were counted in a Neubauer chamber after trypan blue exclusion of dead cells. They were then distributed into wells of a 96-well plate at a final concentration of 2 × 10^6^/ml in RPMI 1640 (Biochrom) enriched with 2 mM glutamine (Biochrom), 10 μg/ml of gentamicin and 100 U/ml of penicillin G. PBMCs were stimulated with 200 μg/ml of highly purified MSU, with 200 μΜ of ultrapure fatty acid [C18:0] which is a TLR2 ligand, with 10 μg/ml of the TLR1/2 ligand palmitoyl-3-cystein [Pam_3_Cys- SKKKK] (EMC MicrocollectionsGmbH, Tübingen, Germany) and with 1 μg/ml of the TLR2/6 ligand fibroblast stimulating ligand-1 [FSL-1] (EMC MicrocollectionsGmbH). Pam_3_CysandFSL-1 stock solutions in distilled water were used. All experiments were run in duplicate with cells of at least one healthy donor per day of experiment. After 24 hours of incubation, plates were centrifuged and supernatants were collected and stored at -80°C until the cytokine assays were performed. For assessment of IL-17 production, the culture period was five days with fetal bovine serum (FBS) added to the culture medium at a final concentration of 10%.

PBMCs used for this analysis consist of lymphocytes and monocytes in an almost 3:1 ratio. Ideally, to measure cytokines released from the monocyte fraction of PBMCs, further monocyte separation should be performed. This generates some danger for *ex vivo *stimulation of monocytes by the separation media providing erroneous false-positive results. However, stimulation of PBMCs for 24 hours provides cytokines coming from the monocyte fraction as already considered in previous publications of our group [16].

### Cytokine measurement

Human TNFα, IL-1β, IL-6, IL-8 and IL-17 in supernatants were measured by ELISA (R&D systems, Minneapolis, MN, USA) according to the instructions of the manufacturer. The lowest limits of detection were 40 pg/ml for TNFα, 20 pg/ml for IL-1β, 16 pg/ml for IL-6, 165 pg/ml for IL-8, and 80 pg/ml for IL-17.

### Quantitative PCR for mRNA expression of IL-1β

PBMCs were cultured with the stimuli as mentioned above and after four hours of incubation at 37°C in 5% CO_2 _the plates were centrifuged, the cell pellets were lysed with 400 μl of Trizol (AppliChem GmbH, Darmstadt, Germany) and kept at -80°C until extraction of RNA.

RNA was extracted with chloroform and gradient centrifugation for 15 minutes at 4°C and 12,000 g followed by treatment for 30 minutes at 37°C with 0.04 U/μl of DNAase (New England BioLabs, Ipswich, MA, USA). RNA was detected after 3% agarose gel electrophoresis and ethidium bromide staining. A total of 1.5 μg of RNA (Pharmacia Biotech photometer) was used for the production of cDNA using 0.4 mM of dNTPs (New England BioLabs), 1 U of RNA-sin (New England BioLabs), 10 mM dithiothreitol (DTT) (AppliChem GmbH) and 5x of the reverse transcriptase buffer in a Sensoquest thermal cycler LabCycler Gradient using appropriate blanks (Eppedorf, Hamburg, Germay). After an initial incubation step of 10 minutes at 60°C, 1 μU of reverse transcriptase (New England BioLabs) was added followed by three cycles: 10 minutes at 25°C, 50 minutes at 42°C and 15 minutes at 70°C. cDNA was kept at -80°C until assayed

Expression of mRNA was tested by the iCycler system (BioRad, Philadelphia, PA, USA) using 1μl of cDNA, 0.1 mg/ml of sense and antisense primers, 3mM of MgCl_2 _(New England BioLabs), 0.25 mM of dNTPs (New England BioLabs), 10x buffer and 1 mM of *Taq *polymerase with SYBR-Gr as a fluorochrome in each tube. Primer sequences were: for IL-1β sense 5'- CAG CTA CGA ATC TCC GAC CAC-3' and antisense 5'- GGC AGG GAA CCA GCA TCT TC-3', and for β_2_-microglobulin sense 5'-ATG AGT ATG CCT GCC GTG TG-3' and antisense 5'-CCA AAT GCG GCA TCT TCA AAC-3'. After an initial denaturation step for 10 minutes at 95°C, 34 cycles were performed. Each cycle consisted of: denaturation for 30 seconds at 95°C, annealing for 30 seconds at 72°C and elongation for 30 seconds at 95°C. Amplification was followed by a melting curve, appropriate blanks were applied. The PCR product was identified after 3% agarose gel electrophoresis and ethidium bromide staining. Quantitative results were expressed as defined by the Pfaffl equation [17], using the efficiency of a standard curve created with known cDNA.

### Statistical analysis

Data on cytokines are expressed as medians ± 95% confidence intervals (CI); those of gene transcripts by their mean ± SE. Comparisons between groups were done using the Mann-Whitney U test. Folds of change of cytokine production after treatment with a combination of stimuli compared with the most active single stimulus were calculated; comparisons between patients and healthy volunteers were done by the Mann-Whitney U test. Any more than a 30% increase was considered as significant synergy. Comparison between groups was done by the Fischer's exact test. Correlations between folds of changes of cytokine production and of the annual number of attacks of acute gouty arthritis of patients were determined according to Spearman. *P *values less than 0.05 were considered significant.

## Results

### Cytokine production after exposure to MSU and TLR ligands

**Figure 2:**
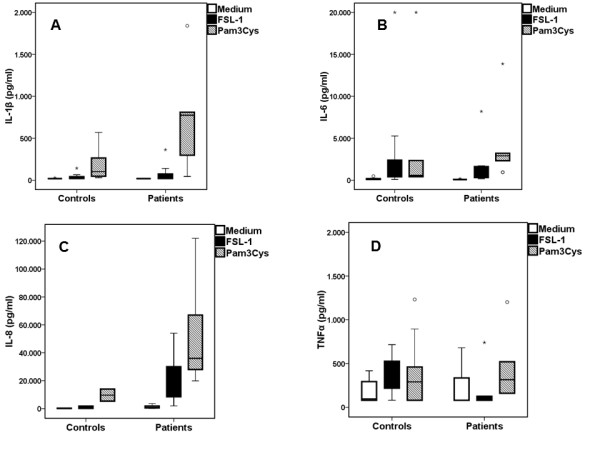
**PBMCs from gout patients produce more IL-1βand IL-6 compared to healthy controls when exposed to TLR ligands**. Release of IL-1β(**A**), IL-6 (**B**), IL-8 (**C**) and TNFα (**D**) by PBMCs of patients with gout and healthy donors after their stimulation with growth medium (RPMI), palmitoyl-3-cystein [Pam_3_Cys] (TLR2/1 agonist) or fibroblast stimulation ligand-1 [FLS-1] (TLR2/6 ligand). Asterisks denote outliers and circles denote extremes. Gout patients n = 18 and healthy controls n = 12. PBMC, peripheral blood mononuclear cells; TLR, toll-like receptor.

We stimulated PBMCs of patients with gout and of healthy donors with either the TLR2/6 ligand FSL-1 or TLR1/2 ligand Pam_3_Cys (Figure 2). We found that production of IL-1β did not significantly differ between patients and controls, although IL-1β production in cells of gout patients showed a wide variation and a tendency to higher production when exposed to Pam_3_Cys (Figure 2A).

**Figure 3:**
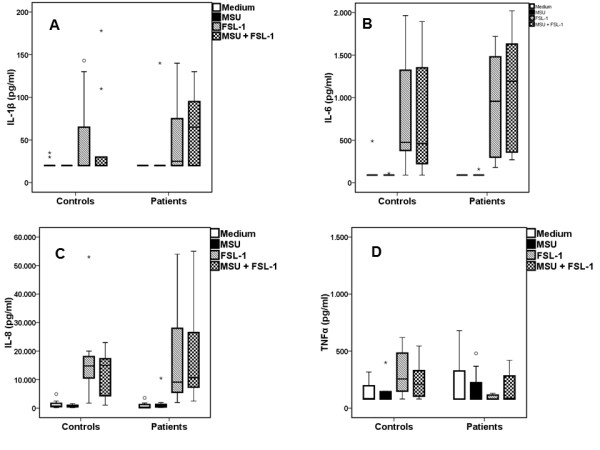
**Lack of synergy between MSU and FSL-1 in the induction of IL-1β**. Release of IL-1β (**A**), IL-6 (**B**) IL-8 (**C**) and TNFα (**D**)by peripheral blood mononuclear cells of patients with gout and healthy donors after having been left untreated (RPMI) or stimulated by monosodium urate monohydrate (MSU) crystals alone or in combination with fibroblast stimulation ligand-1 [FLS-1]. Asterisks denote outliers and circles denote extremes. *P *values indicate significant differences between respective values of patients and healthy controls by Mann-Whitney U-test. Gout patients n = 18 and healthy controls n = 12.

**Figure 4:**
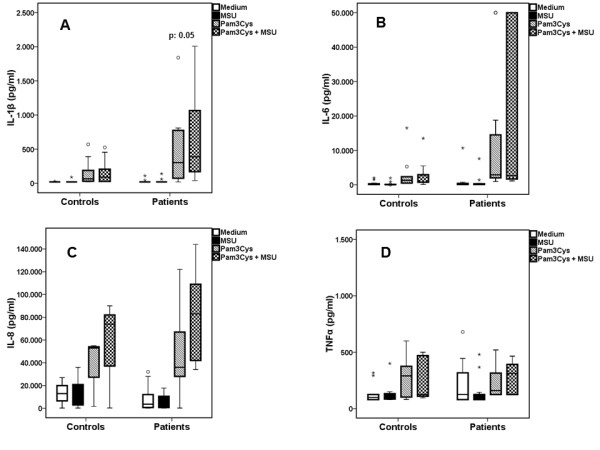
**Synergy between MSU and Pam_3_Cys in the induction of IL-1β was greater in patients than controls**. Release of IL-1β (**A**), IL-6 (**B**), IL-8 (**C**) and TNFα (**D**) by peripheral blood mononuclear cells of patients with gout and healthy donors after having been left untreated (RPMI) or stimulated by monosodium urate monohydrate (MSU) crystals alone or in combination with palmitoyl-3-cystein [Pam_3_Cys]. Asterisks denote outliers and circles denote extremes. *P *values indicate significant differences between respective values of patients and healthy controls by Mann-Whitney U-test. Gout patients n = 18 and healthy controls n = 12.

Exposure of PBMCs to ultrapure MSU crystals did not induce IL-1β production in either of the two groups (Figure 3A). Co-incubation of MSU with FSL-1 did not change the release of cytokines achieved by single FSL-1 (Figure 3). When PBMCs of gout patients were incubated with MSU crystals together with Pam3Cys, production of IL-1β was significantly increased compared to Pam3Cys alone (Figure 4). IL-6, IL-8 or TNFα released by PBMCs was not significantly different between gout patients and controls for any of the stimuli, single or in combination (Figures 2, 3 and 4).

### Cytokine induction by MSU and saturated fatty acids

**Figure 5:**
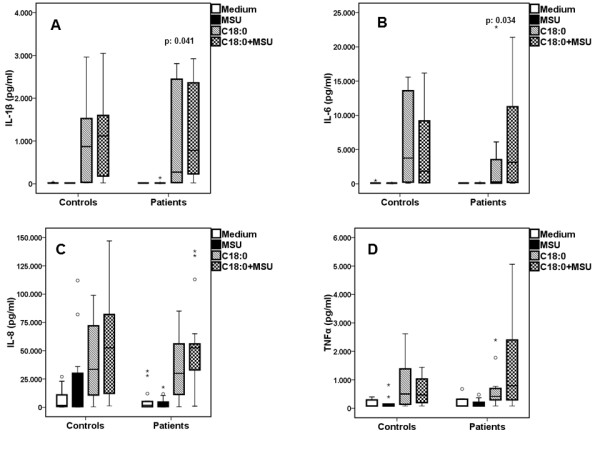
**Patients displayed greater synergy between MSU and C18:0 in the induction of IL-1β than healthy donors**. Release of IL-1β (**A**), IL-6 (**B**), IL-8 (**C**) and TNFα (**D**)by peripheral blood mononuclear cells of patients with gout and healthy volunteers after stimulation with medium alone (RPMI), monosodium urate monohydrate (MSU) crystals and saturated fatty acids C18:0 alone or in combination with MSU. Asterisks denote outliers and circles denote extremes. *P *values indicate significant differences between respective values of patients and healthy controls by Mann-Whitney U test. Gout patients n = 18 and healthy controls n = 12.

Recently, we demonstrated that the addition of C18:0 fatty acid augments IL-1β production of PBMCs exposed to MSU [12]. In the present study, we show that PBMCs from gout patients produce more IL-1β than controls when incubated with C18:0 and MSU (Figure 5A). However, we noted that exposure of PBMCs to C18.0 alone also stimulated IL-1β production, most likely due to a different preparation of C18.0 stimuli than in previous studies (containing a low final concentration of 0.55% ethanol). The production of IL-6 by MSU/C18:0 was in line with IL-1β production in cells isolated from gout patients, with a trend towards higher cytokine production in gout patients than in healthy controls. Release of TNFα and of IL-8 after co-stimulation with C18:0 and MSU did not differ between patients and healthy controls.

**Figure 6:**
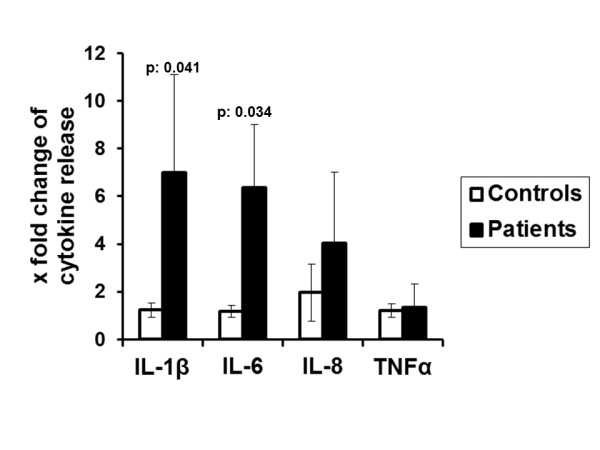
**Changes of production of cytokines from PBMCs of gout patients and of healthy volunteers after stimulation with FFA C18:0 and MSU**. x fold refers to changes compared with the most active single stimulus. *P *values indicate significant differences between respective values of patients and healthy controls by Mann-Whitney U-test. Gout patients n = 18 and healthy controls n = 12. FFA, free fatty acids; MSU, monosodium urate monohydrate; PBMC, peripheral blood mononuclear cells.

**Figure 7:**
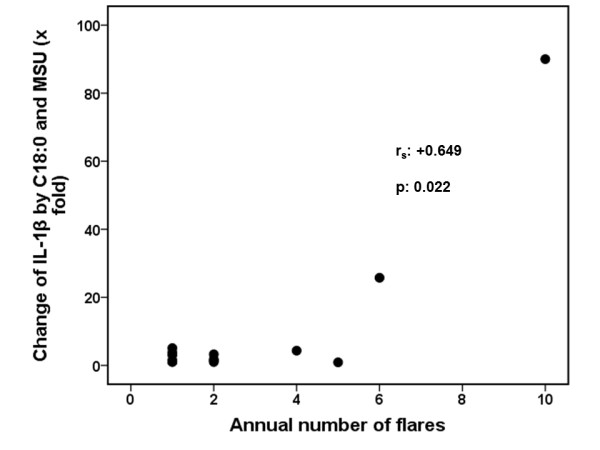
**Correlation between synergy for IL-1β production and the annual number of attacks of acute gouty arthritis**. The x fold change of IL-1β produced by the combination of free fatty acids C18:0 and monosodium urate (MSU) compared with C18:0 alone is shown.

Undoubtedly, the wide variation in cytokine production raises questions about the robustness of the finding. In order to make the result robust four types of analysis were performed: a) statistical comparisons reported in Figures 5A and 5B were performed using non-parametric tests; b) qualitative analysis disclosed that synergy of FFA C18:0 with MSU crystals for the production of IL-1β and IL-6 was found in 15 of 18 patients versus 2 of 12 controls, *P *= 0.001); c) fold-changes of IL-1β and of IL-6 production after stimulation of PBMCs of patients with the combination of C18:0 and MSU crystals compared to C18:0 alone were significantly greater compared to respective changes of healthy controls (Figure 6); and d) a positive correlation was found between the change of IL-1β production after stimulation of PBMCs of patients with the combination of C18:0 and MSU crystals compared to C18:0 alone and the annual number of attacks of acute gouty arthritis of these patients (*P = *0.022, Figure 7). Significant correlations were not found between changes of IL-1β by the combination of Pam3Cys and MSU compared to Pam3Cys alone and the annual number of attacks of acute gouty arthritis (data not shown, r_s_: +0.596, *P = *0289).

To exclude the possibility that part of the explanation for the synergy between C18:0 and MSU is due to the younger age of healthy controls, correlation analysis was done between the age of both healthy donors and patients and the fold change of IL-1β production by the combination compared with C18:0 alone. No statistically significant results were found (r_s_: +0.170, *P *= 0.428).

### Lack of IL-17 production by PBMCs after MSU and TLR2 ligands

A hallmark of acute gout is the influx of neutrophils into the synovium and joint fluid [18]. In order to define over-production in IL-1β release as the major culprit for attacks of gout, production of other cytokines mediating chemotaxis of neutrophils, notably IL-8 and IL-17, should not be affected by co-incubation with C18:0 and MSU. The cytokine IL-17 induces neutrophil-attracting chemokines and granulopoietic cytokines, and recruits neutrophils to the site of inflammation [19]. PBMCs of both gout patients and healthy controls exhibited no detectable production of IL-17 after stimulation with MSU crystals for five days, either in the presence or absence of FSL-1, Pam3Cys or C18:0 fatty acids (data not shown).

### Effect on gene transcription

**Figure 8:**
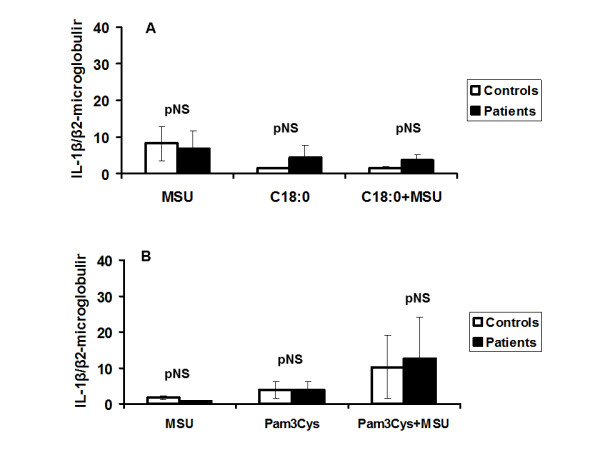
**Gene expression of IL-1β in PBMCs from gout patients stimulated with MSU and Pam3Cys compared with healthy controls**. Measurement of mRNA transcripts of IL-1β after stimulation of PBMCs of patients with gout and controls with FFA C18:0 (**A**) or Pam_3_Cys (**B**) alone or in combination with monosodium urate (MSU) crystals. Results are shown as ratios with the transcripts of β_2_- microglobulin. Gout patients n = 8 and healthy controls n = 6. FFA, free fatty acids; MSU, monosodium urate monohydrate; Pam_3_Cys, Pam_3_Cys, palmitoyl-3-cystein; PBMC, peripheral blood mononuclear cells.

The synergy between TLR-2 ligands and MSU crystals might lead either to enhanced expression of the IL-1β gene or to increased cleavage of pro-IL-1β by caspase-1. mRNA transcripts of IL-1β in the PBMCs of gout patients did not have any difference after addition of MSU crystals to FFA C18:0, compared with healthy donors (Figure 8A). Similar findings were observed after stimulation with MSU and Pam3Cys (Figure 8B).

## Discussion

In the present paper, we report that PBMCs of gout patients who have experienced attacks of arthritis produce more IL-1β than PBMCs of healthy volunteers when the cells are exposed to a combination of TLR2 ligands and MSU crystals. In addition, the IL-1β response in the presence of C18:0 fatty acids together with MSU is augmented in the majority of the gout patients.

The responses in the gout patients show a much wider variation than those in healthy volunteers. These variations may be partly explained by the variations of the annual attacks of acute gouty arthritis in the patient group since synergy for IL-1β production is pronounced for patients with frequent attacks of acute gouty arthritis. We found that the synergism between MSU and FFA C18:0 leading to production of IL-1β is not due to enhanced gene expression. The most likely explanation is increased cleavage of pro-IL-1β to IL-1β in patients with gout. Enhanced cleavage may be mediated through the NLRP3 inflammasome, since MSU is one agonist for the NLRP3 inflammasome. Our finding that this synergy was also present for IL-6 (which is at least in part induced by endogenous bioactive IL-1) underscored the activation of the inflammasome. However, it should not be neglected that other proteinases, namely, neutrophil elastase and cathepsin G, may mediate cleavage of pro-IL-1β to IL-1β [20] but this is unlikely in the present study since they are present at very low levels in the PBMC preparations.

Our findings suggest that the NLRP3 inflammasome may be activated more readily in patients with gout than in healthy controls. The synergistic effects for the production of IL-1β were mainly found between MSU and Pam3Cys and between MSU and FFA C18:0, but not between MSU and FSL-1, providing evidence that MSU synergizes with TLR2/1 agonists but not with TLR2/6.

Several groups have shown that MSU crystals function as inflammasome activators [9-11]. Initial reports suggested that urate crystals were able to induce IL-1β on their own. When we reported that MSU crystals need the presence of a second stimulus such as LPS [11] or FFA C18:0 [12], it turned out that others had also used LPS as an inducer of pro-IL-1β transcripts [13]. This would fit with everyday clinical practice: while hyperuricemia is a prerequisite for gout, its presence does not always lead to disease and a second stimulus such as infection, excessive alcohol intake or a fatty meal is needed for an attack to occur. The data in the literature on IL-1β production in gout were obtained by the use of either primary mononuclear cells of healthy volunteers [9,11,12] or human monocytic cell lines (THP-1) cells [10]; this is the first study using cells of gout patients.

The infiltration of the synovium by neutrophils is considered to be a feature of the acute inflammation in gout [1]. The recent literature indicates that IL-17 is a potent mediator of neutrophil influx, by expansion of the neutrophil lineage through induction of Granulocye-colony stimulating factor (G-CSF) as well as by recruitment of neutrophils through the induction of chemokines [19]. IL-17 is mainly released by a distinct subset of T helper cells, called Th17, which derive from the naïve CD4^+ ^T-cells [21]. We measured IL-17 in order to find out whether acute gouty inflammation is under the control of IL-17. The lack of production of IL-17, observed in the present study, supports the view that neutrophil influx in gouty inflammation is probably due to the release of other neutrophil-recruiting cytokines, such as IL-1β. In fact, Th17 cells are generated after days and this is not compatible with the rapid occurrence of inflammation in gout.

Despite our inability to find a direct relationship between uric acid concentrations in serum and the degree to which the inflammasome is activated, hyperuricemia still may be the explanation for the increased activation of the inflammasome in patients with gout. At the molecular level uric acid, in its soluble form, has been reported to have a role in regulating redox homeostasis by forming radicals in reaction with other oxidants [22]. Moreover, it has been suggested that reactive oxygen species (ROS) may activate the NLRP3 inflammasome [23]. However, based on the findings of a previous study by our group, ROS are not involved in inflammasome activation, since PBMCs from ROS deficient patients produce significantly more IL-1β after exposure to MSU crystals than the cells from healthy individuals [24].

Our findings were generated with circulating PBMCs of patients. As such, it may be speculated that the over-inflammatory state produced in gout is systemic and not limited to joints. This speculation is consistent with the wide variation of co-morbidities recognized in these patients [1].

## Conclusions

The present study provides evidence that the PBMCs of patients with gout display a more prominent synergistic production of IL-1β after stimulation with MSU crystals and TLR-2/1 ligands, compared to healthy controls. This synergy may be mediated by enhanced maturation of pro-IL-1β into IL-1β.

## Abbreviations

CHD: coronary heart disease; CI: confidence interval; COPD: chronic obstructive pulmonary disease; CRD: chronic renal disease; DCs: dendritic cells; DM2: diabetes mellitus type 2; ELISA: enzyme-linked immunosorbent assay; FBS: fetal bovine serum; FFA: free fatty acids; FSL-1: fibroblast stimulating ligand-1; IL interleukin; LPS: lipopolysaccharide; MSU: monosodium urate monohydrate; Pam_3_Cys: palmitoyl-3-cystein; PBMCs: peripheral blood mononuclear cells; PBS: phosphate-buffered saline; PCR: polymerase chain reaction; SE: standard error of the mean; TLR: toll-like receptors; TNFα: tumor necrosis factor alpha; ROS:reactive oxygen species; VH: vascular hypertension.

## Competing interests

The authors declare that they have no competing interests.

## Authors' contributions

EM and MM participated in the acquisition of data, analysis and interpretation and the preparation of the manuscript. TOC, SM, AP, MG and AS contributed to the acquisition of data. EG-B, MN, JvdM and LJ participated in study design, analysis and interpretation of data and manuscript preparation. All authors approved the final version of the article.
